# Leishmaniasis in Norway Rats in Sewers, Barcelona, Spain 

**DOI:** 10.3201/eid2506.181027

**Published:** 2019-06

**Authors:** Maria Teresa Galán-Puchades, Mercedes Gómez-Samblás, Jose M. Suárez-Morán, Antonio Osuna, Joan Sanxis-Furió, Jordi Pascual, Rubén Bueno-Marí, Sandra Franco, Víctor Peracho, Tomás Montalvo, Màrius V. Fuentes

**Affiliations:** Universitat de València, Burjassot-Valencia, Spain (M.T. Galán-Puchades, J. Sanxis-Furió, M.V. Fuentes);; Universidad de Granada, Granada, Spain (M. Gómez-Samblás, J.M. Suárez-Morán, A. Osuna);; Laboratorios Lokímica, Catarroja-Valencia, Spain (J. Sanxis-Furió, R. Bueno-Marí);; Agència de Salut Pública de Barcelona, Barcelona, Spain (J. Pascual, S. Franco, V. Peracho, T. Montalvo);; CIBER Epidemiology and Public Health, Barcelona (T. Montalvo)

**Keywords:** Leishmania infantum, Rattus norvegicus, Barcelona, Spain, reservoir, sewage system, parasites, leishmaniasis

## Abstract

We detected *Leishmania infantum* in 98 Norway rats (*Rattus norvegicus*) trapped in parks and sewers of Barcelona, Spain. The 84 rats from the sewers showed a prevalence of 33.3% and up to 2,272 estimated parasites. These results, in the most abundant potential reservoir in cities, is of public health concern.

Canine and human leishmaniasis caused by *Leishmania infantum* is considered an emerging disease in the Mediterranean basin (*1*). In addition to dogs, several wild mammals have been found infected by *L. infantum* in rural environments in Europe (*2*). With regard to the epidemiologic factors promoting infection with *Leishmania*, the appearance of new animal reservoirs besides dogs has been highlighted (*1*). In this context, only a few studies examine the possible reservoir role of synanthropic animals in cities, where the role of certain domestic mammals has been analyzed exclusively (*1*).

Because human leishmaniasis is endemic in Barcelona, Spain (*3*), we investigated and quantified the presence of *L. infantum* in an urban population of the Norway rat, *Rattus norvegicus,* using a highly sensitive quantitative PCR (qPCR) method for *Leishmania* DNA detection. Rat leishmaniasis could complicate the epidemiologic situation of human and canine leishmaniasis, considering that the Norway rat is the most widespread mammal in the world after humans and also the most abundant animal in cities.

We trapped 98 Norway rats, 84 in the sewage system and 14 in parks, during the winter of 2016–17 in a rodent surveillance and control program in Barcelona (permission no. SF/044 obtained from the regional government of Catalonia). We treated the rats according to Directive 2010/63/EU of the European Parliament and Council decision of September 22, 2010. We obtained DNA from 10 mg of spleen using the Purification of Total DNA Kit (QIAGEN, https://www.qiagen.com), following the manufacturer’s instructions. We processed the samples whose DNA concentration was too low with the extraction kit by the phenol–chloroform–isoamyl (25:24:1) DNA extraction technique. We quantified the parasite DNA by qPCR using Taqman probe with Fam fluorochrome (*4*).

Only 1 rat (7.1%) captured in the parks tested positive for *L. infantum*. However, rats captured in the sewage system showed a 33.3% prevalence (28/84) of *L. infantum* infection. The estimated number of parasites in the positive samples from spleens varied considerably, ranging from 0.28 to >2,200 (Table). Histologic sections of positive spleens were stained by Giemsa and by the streptavidin–biotin peroxidase complex immunohistochemical method (*5*) (Appendix Figure). 

**Table:** Real-time PCR results for the 29 *Leishmania infantum*–positive Norway rats analyzed, Barcelona, Spain*

Rat no.	C_t_†	Estimated no. parasites	SD
1	32.81	75.67	25.70
2	32.33	50.68	12.21
3	29.25	723.15	345.67
4	30.84	344.30	56.40
5	38.50	0.28	0.20
6	28.49	10.81	3.36
7	28.46	2,272.46	771.72
8	32.32	118.12	63.76
9	29.60	516.20	199.36
10	28.10	14.24	2.98
11	30.19	566.28	7.32
12	28.41	11.98	5.09
13	29.96	679.46	35.59
14	31.47	31.38	0.20
15	31.46	215.66	66.84
16	30.79	356.32	42.03
17	33.01	66.31	31.43
18	29.68	465.21	68.73
19	30.75	370.40	70.72
20	31.60	1.44	1.51
21	26.52	48.81	20.15
22	31.82	159.43	27.73
23	28.15	0.33	17.66
24	28.56	9.98	1.30
25	30.25	547.67	93.15
26	30.93	161.73	11.80
27	28.38	11.59	2.70
28	29.84	761.38	223.52
29	33.16	55.22	1.41

*C_t_, cycle threshold. †Mean values of the 3 replicates of each sample. Cutoff established: positive result, C_t_ <35; negative result, C_t_ ≥35.

The low number of infected Norway rats found in Mediterranean countries in Europe so far has led to the species being categorized as an incidental host, capable of becoming infected but considered irrelevant to the long-term persistence of the disease (*2*,*6**–**8*). However, our study demonstrates the importance of the trapping site for finding a large *Leishmania*-infected rat population. 

According to the World Health Organization, the incrimination of a particular mammal as a *Leishmania* reservoir must depend on an accumulation of evidence (*9*). First, the reservoir must be sufficiently abundant and long-lived. In this sense, the Norway rat is the most abundant mammal in cities, with a lifespan of around 1–3 years. The lack of predators or interspecific competition in cities guarantees a longer lifetime than in wild environments. Second, intense host–sand fly contact is necessary. Sewers are a breeding site for *Phlebotomus* sand flies, which can reach abundant population levels (*10*). Third, the prevalence of *Leishmania* infection should be >20%. Our study revealed a 33.3% prevalence in Norway rats in the sewers. Fourth, the course of infection should be nonpathogenic and long enough to enable the parasites to survive any nontransmission season. Neither splenomegaly nor hepatomegaly were evident in the affected animals, although further studies should be addressed to assess the true pathogenic degree of rat leishmaniasis. Finally, parasites should be available in the skin or the blood in sufficient numbers to be taken up by a sand fly. The availability of the parasite to the sand fly cannot be proved by our findings. However, in naturally infected Norway rats in Spain, *L. infantum* was detected in the hair of the rats by molecular methods (*7*).

Only counting sewer rats, and not those living above ground, a 0.13 rat-per-person scenario is suspected for Barcelona (Agència de Salut Pública de Barcelona, pers. comm., June 2018). Therefore, the prevalence found in the sewers in this study means that there could be >70,000 underground rats with leishmaniasis in Barcelona (1,620,809 human inhabitants in 2017), a figure of public health concern for a potential reservoir.

If future xenodiagnostic studies determine that sand flies become infected successfully by the rats, as has already been demonstrated in the case of its congener, the black rat, (*R. rattus*) (*11*), the finding of a new reservoir, besides the dog, would be a major advance in the epidemiology of leishmaniasis. However, the complexity of rat control would represent a great hindrance to overcome.

Appendix Histological sections of naturally infected rat spleens with *Leishmania*.
