# Supplementary material for: Leishmaniasis in Norway Rats in Sewers, Barcelona, Spain
Source: Emerg Infect Dis. 2019 Jun;25(6):1222–4. doi: 10.3201/eid2506.181027 (PMC6537722; doi:10.3201/eid2506.181027)
Supplement: Appendix — Histological sections of naturally infected rat spleens with Leishmania. [file 18-1027-Techapp-s1.pdf]

Article DOI: https://doi.org/10.3201/eid2506.181027

Leishmaniasis in Norway Rats in Sewers, Barcelona, Spain

Appendix

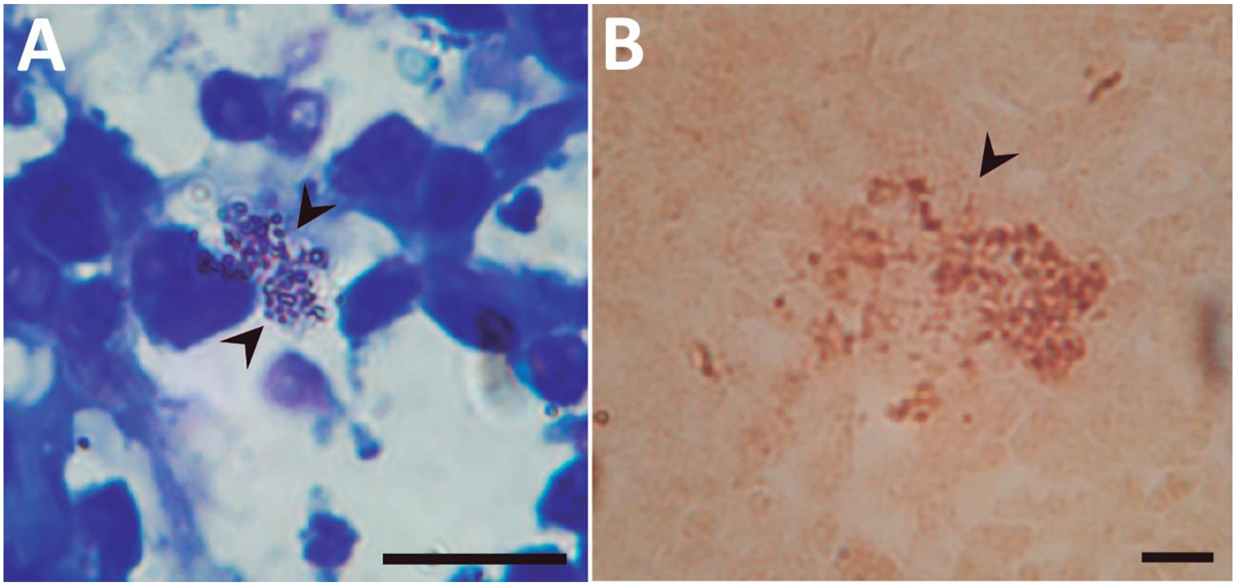

Appendix Figure. Histological sections of naturally infected rat spleens with *Leishmania* (arrows). A) Giemsa stain. B) Immunolabeled amastigotes by the streptavidin–biotin peroxidase complex method (*1*). Scale bars represent 10 μm.

Reference

1. Tafuri WL, Santos RL, Arantes RM, Gonçalves R, de Melo MN, Michalick MS, et al. An alternative immunohistochemical method for detecting *Leishmania* amastigotes in paraffin-embedded canine tissues. J Immunol Methods. 2004;292:17–23. http://dx.doi.org/10.1016/j.jim.2004.05.009
